# Development and Identification of a Novel Anti-HIV-1 Peptide Derived by Modification of the N-Terminal Domain of HIV-1 Integrase

**DOI:** 10.3389/fmicb.2016.00845

**Published:** 2016-06-10

**Authors:** Marina Sala, Antonia Spensiero, Francesca Esposito, Maria C. Scala, Ermelinda Vernieri, Alessia Bertamino, Michele Manfra, Alfonso Carotenuto, Paolo Grieco, Ettore Novellino, Marta Cadeddu, Enzo Tramontano, Dominique Schols, Pietro Campiglia, Isabel M. Gomez-Monterrey

**Affiliations:** ^1^Department of Pharmacy, University of SalernoSalerno, Italy; ^2^Department of Life and Environmental Sciences, Cittadella Universitaria di Monserrato, University of CagliariCagliari, Italy; ^3^Department of Sciences, University of BasilicataPotenza, Italy; ^4^Department of Pharmacy, Medicicnal Chemistry and Toxicologic, University of Naples Federico IINapoli, Italy; ^5^Institute of Genetic and Biomedical Research, National Research Council, Citadella di MonserratoCagliari, Italy; ^6^Department of Microbiology and Immunology, Rega Institute for Medical ResearchLeuven, Belgium

**Keywords:** HIV-1, integrase, N-terminal domain, peptides, inhibitors

## Abstract

The viral enzyme integrase (IN) is essential for the replication of human immunodeficiency virus type 1 (HIV-1) and represents an important target for the development of new antiretroviral drugs. In this study, we focused on the N-terminal domain (NTD), which is mainly involved into protein oligomerization process, for the development and synthesis of a library of overlapping peptide sequences, with specific length and specific offset covering the entire native protein sequence NTD IN 1–50. The most potent fragment, VVAKEIVAH (peptide **18**), which includes a His residue instead of the natural Ser at position 39, inhibits the HIV-1 IN activity with an IC_50_ value of 4.5 μM. Amino acid substitution analysis on this peptide revealed essential residues for activity and allowed us to identify two nonapeptides (peptides **24** and **25**), that show a potency of inhibition similar to the one of peptide **18**. Interestingly, peptide **18** does not interfere with the dynamic interplay between IN subunits, while peptides **24** and **25** modulated these interactions in different manners. In fact, peptide **24** inhibited the IN-IN dimerization, while peptide **25** promoted IN multimerization, with IC_50_ values of 32 and 4.8 μM, respectively. In addition, peptide **25** has shown to have selective anti-infective cell activity for HIV-1. These results confirmed peptide **25** as a hit for further development of new chemotherapeutic agents against HIV-1.

## Introduction

Integrase (IN) is a key enzyme for the integration of the HIV-1 genome into the host cell chromosome and, therefore, a very promising target for anti-AIDS drug design. IN performs two essential catalytic reactions. The first one takes place in the cytoplasm within particles termed preintegration complex (PIC). IN cleaves the conserved dinucleotides GT from the 3′ ends (3′-processing) of the double-stranded viral DNA, generating reactive 3′-hydroxyl DNA ends. Following translocation to the nucleus, IN uses the hydroxyl ends as a substrate reaction for a nucleophilic attack integrating the viral genome in the host chromosomal DNA (strand-transfer reaction, STIN) (Semenova et al., 2006; Delelis et al., 2008).

IN is a protein of 32 kDa organized in three domains (Wang et al., 2001; Chiu and Davies, 2004). The N-terminal domain (NTD, residues 1–50) contains a zinc-binding H^12^H^16^C^40^C^43^ motif and contributes to protein oligomerization process (Engelman and Craigie, 1992; Cai et al., 1997). The central domain (CCD, residues 51–212) encloses the enzyme active site characterized by the catalytic triad D^64^D^116^E^152^, which is highly conserved in all INs, with the two residues D^64^ and D^116^ forming a coordination complex with divalent ions like Mn^2+^ and Mg^2+^ (Goldgur et al., 1998; Chen et al., 2000). The C-terminal domain (CTD, residues 213–288), which is the least conserved domain between retroviral INs, binds non-specifically to DNA and is also involved in IN multimerization (Chen et al., 2000).

Despite the CCD contains the catalytic site, in the absence of the NTD and CTD domains, IN can only catalyze the disintegration reaction *in vitro*, while it needs both the NTD and CTD in a dimeric complex to catalyze 3′-processing and strand-transfer reactions (Lia et al., 2011). Until now, only inhibitors targeting the catalytic site of IN with a specific effect on strand-transfer process (INSTIs) have been identified and developed (Pommier et al., 2005; Savarino, 2006; Dayam et al., 2008; Katlama and Murphy, 2012; Esposito and Tramontano, 2013). In 2007, the INSTI Raltegravir became the first IN inhibitor approved for use in the treatment of HIV infected patients (Evering and Markowitz, 2007; Summa et al., 2008). More recently, the FDA also approved Stribild, a single-tablet regimen HIV medication containing four drug combination of Elvitegravir, another HIV INSTI, Cobicistat, a CYP3A inhibitor, and Emtricitabine and Tenofovir DF, both HIV nucleoside analog reverse transcriptase inhibitors (Shimura et al., 2008; Brinson, 2013).

The emergence of the resistance to these drugs gives raise to the pressing need of novel IN inhibitors (Mesplède et al., 2012). However, absence of information on the structures of a full-sized enzyme, on its complex with DNA and also on the PIC composition and operation complicates and slows the search for novel IN inhibitors.

To overcome these drawbacks, targeting allosteric sites of the protein including interaction site of IN with cellular co-factor essential for integration, for example the LEDGF/p75-IN interaction, or oligomerization/multi oligomerization sites might represent alternative approaches to IN inhibition (Zhao et al., 2003; Li et al., 2006; Al-Mawsawi and Neamati, 2011; Maes et al., 2012; Christ and Debyser, 2013; Long et al., 2013; Di Santo, 2014).

In this context, compounds that target the IN N-terminal domain would be effective at disrupting IN function through a range of mechanisms, including allosteric or oligomerization inhibition. We considered this domain an important starting point for the identification of peptide inhibitors in order to clarify some points on IN function in viral replication, such as the elucidation of HIV-1 IN polymerization state, or its potentiality in the structure-based design.

Here we present the design and synthesis of peptides targeting the integration events by directly inhibiting IN or its multimerization process. The synthesized peptides were assayed *in vitro* for their ability to inhibit IN strand transfer activity and for the capability to inhibit IN dimerization or to promote IN multimerization. Finally, the most potent compounds, conveniently conjugated with cell-penetrating fragment Tat, were assayed in MT-4 cells for determining anti-HIV infective activity.

## Materials and methods

N^α^-Fmoc-protected amino acids, Rink amide-resin, HOAt, HOBt, HBTU, DIEA, piperidine, and trifluoroacetic acid were purchased from Iris Biotech (Germany). Rink Amide-ChemMatrix resin was purchased from Biotage AB (Sweden). Peptide synthesis solvents, reagents, as well as CH_3_CN for HPLC were reagent grade and were acquired from commercial sources and used without further purification unless otherwise noted.

### Peptide synthesis

The synthesis of IN analogs was performed according to the solid phase approach using standard Fmoc methodology in a manual reaction vessel and automated microwave synthesizer (Wang et al., 1989; Malik et al., 2010). The first amino acid, N^α^-Fmoc-Xaa-OH (N^α^-Fmoc-Asp(OtBu)-OH, N^α^-Fmoc-Glu(OtBu)-OH, N^α^-Fmoc-His(N(_im_)trityl(Trt))-OH, N^α^-Fmoc-Trp(Boc)-OH, N^α^-Fmoc-Ala-OH, N^α^-Fmoc-Leu-OH, N^α^-Fmoc-Val-OH, N^α^-Fmoc-Lys(Boc)-OH, N^α^-Fmoc-Cys(Trt)-OH, N^α^-Fmoc-Met-OH, N^α^-Fmoc-Ser(tBu)-OH, was linked on to the Rink resin (100–200 mesh, 1% DVB, 0.59 mmol/g) previously deprotected by a 25% piperidine solution in DMF for 30 min. The following protected amino acids were then added stepwise. Each coupling reaction was accomplished using a three-fold excess of amino acid with HBTU (3 eq.) and HOBt (3 eq.) in the presence of DIEA (6 eq.). The N^α^-Fmoc protecting groups were removed by treating the protected peptide resin with a 25% solution of piperidine in DMF (1 × 5 min and 1 × 25 min). The peptide resin was washed three times with DMF and the next coupling step was initiated in a stepwise manner. The peptide resin was washed with DCM (3 ×), DMF (3 ×), and DCM (3 ×), and the deprotection protocol was repeated after each coupling step. In addition, after each step of deprotection and after each coupling step, Kaiser test was performed to confirm the complete removal of the Fmoc protecting group, respectively, and to verify that complete coupling has occurred on all the free amines on the resin. The N-terminal Fmoc group was removed as described above, and the peptides were acetylated adding a solution of Ac_2_O/DCM (1:3) shaking for 30 min. Finally the peptides were released from the resin with TFA/iPr_3_SiH/H_2_O (90:5:5) for 3 h. The resin was removed by filtration, and the crude peptide was recovered by precipitation with cold anhydrous ethyl ether to give a white powder and then lyophilized.

### Microwave peptides synthesis

The peptides **Tat-18**, **Tat-24**, and **Tat-25** were synthesized using a Biotage Syro Wave fully automated microwave and parallel peptide synthesizer or assembled on the Automated Microwave Peptide Synthesizer from Biotage AB (Initiator + Alstra^TM^).

Peptides were synthesized on a Rink Amide-ChemMatrix resin (150 mg, loading 0.4–0.6 mmol/g), previously deprotected with 25% piperidine/DMF (1 × 3 min, 1 × 10 min) at room temperature. The resin was then washed with DMF (4 × 4.5 ml). The following protected amino acids were then added on to the resin stepwise. Coupling reactions were performed using N^α^-Fmoc amino acids (3.0 eq., 0.5 M), using as coupling reagent HBTU (3eq, 0.6 M), HOAt (3eq, 0.5 M), and DIEA (6eq, 2 M) in N-methyl-2-pyrrolidone (NMP). All couplings were achieved for 10 min at 75°C (2 ×) and 2 × 45 min at RT for histidine and cysteine couplings to avoid the epimerization. After each coupling step, the Fmoc protecting group was removed as described above. The resin was washed with DMF (4 × 4.5 ml) after each coupling and deprotection step. Finally peptides were released as described above.

### Purification and characterization

All crude peptides were purified by RP-HPLC on a preparative C18-bonded silica column (Phenomenex Jupiter 100 proteo 90 Å, 100 × 21.20 mm, 10 μm) using a Shimadzu SPD 10A UV−Vis detector, with detection at 210 and 254 nm. The column was perfused at a flow rate of 15 mL/min with solvent A (10%, v/v, water in 0.1% aqueous TFA), and a linear gradient from 10 to 90% of solvent B (80%, v/v, acetonitrile in 0.1% aqueous TFA) over 25 min was adopted for peptide elution. Analytical purity, retention time (tR) and peptides molecular weights were determined by ESI mass spectrometry in Shimadzu a LC-MS 2010 instrument fitted with Phenomenex, Aeris XB-C18 column (150 mm × 4.60, 3.6 μm).

### IN activity inhibition assay

Express Biotech International Company performed this assay using its HIV-1 IN Assay kit (Xpressbio Life Science Products, USA. www.xpressbio.com). Briefly, Streptavidin coated 96-well plates were coated with a double-stranded HIV-1 LTR U5 donor substrate (DS) oligonucleotide containing an end-labeled biotin. Full-length recombinant HIV-1 integrase protein was then loaded onto this oligo substrate. Compounds were added to the reaction and then a different double-stranded target substrate (TS) oligo containing 3′-end modifications was added to the plate. The sequence of DS DNA and TS DNA substrates used in this assay were

5′-P ACC CTT TTA GTC AGT GTG GAA AAT CTC TAG
CAG T
GAA AAT CAG TCA CAC CTT TTA GAG ATC GTC A
and
5′ TGA CCA AGG GCT AAT TCA CT
ACT GGT TCC CGA TTA GAS GA

respectively (Hazuda et al., 1994 and Engelman et al., 1993). The HIV-1 IN cleaves the terminal two bases from the exposed 3′-end of the HIV-1 LTR DS and then catalyzes a strand-transfer reaction to integrate the DS into the TS. The products of the reaction were detected colorimetrically using an HRP-labeled antibody directed against the TS 3′-end modification. Percent inhibition in the IN assay was calculated and IC_50_ values were determined.

### HTRF-based IN subunit exchange assay

Full-length IN and LEDGF proteins were expressed in *E. coli* BL21(DE3) and were purified as described (Kessl et al., 2012; Esposito et al., 2015; Tintori et al., 2015). His- and FLAG-tagged INs, were mixed in 25 mM Tris (pH 7.4) buffer containing 150 mM NaCl, 2 mM MgCl_2_, 0.1% Nonidet P-40, 1 mg/ml BSA. Test compounds were then added to the mixture. A mixture of anti-His6-XL665 and anti-FLAG-EuCryptate antibodies were then added to the reaction and, after an incubation, the plate are read and the HTRF signal is calculated from the 665/620 nm ratio.

### Circular dichroism (CD)

All CD spectra were recorded using a JASCO J710 spectropolarimeter at 20°C between λ = 260–190 nm (1 mm path, 1 nm bandwidth, 4 accumulations, and 100 nm min^−1^ scanning speed). Measurements were performed with peptides in H_2_O (0.100 mM, pH 7.4) or in in 50% TFE/water solution.

### Cell-based assays

The HIV-1 strain NL4.3 was obtained from the AIDS Research and Reference Reagent Program (Division of AIDS, NIAID, NIH, USA). The HIV-2 strain ROD was obtained from the Medical Research Council (MRC, London, UK). The anti-HIV assay in MT-4 cells has been described previously (Vermeire et al., 2004). MT-4 cells (1 × 10^6^ cells/ml; 50 μl of volume) were pre-incubated for 30 min at 37°C with the test compounds in 96-well plates (Falcon, BD Biosciences). The laboratory HIV strains (NL4.3 and ROD) were added according to the 50% tissue culture infectious dose (TCID50) of the viral stock. Cellular cytopathicity was scored microscopically in MT-4 cells 5 days post-infection and IC_50_s were calculated spectrophotometrically using MTS/PES. For the latter viability assay, the Cell-Titer96 Aqueous One Solution Proliferation Assay (Promega, Leiden, The Netherlands) kit was used.

## Results

### Design

**Figure 1:**
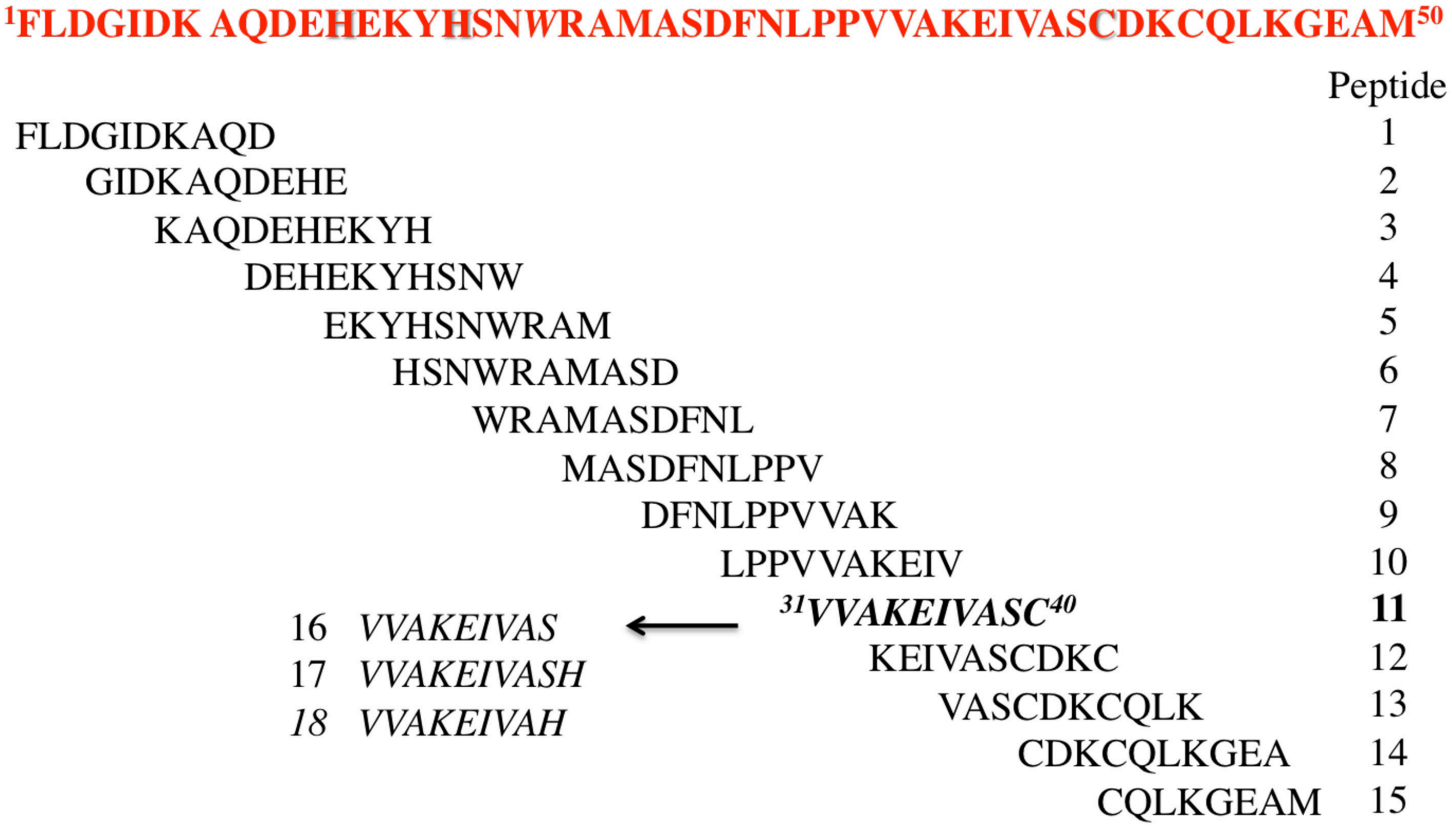
**Sequences of the designed peptides in this study**.

Starting from HIV-1 IN NTD we synthesized an overlapping peptide library to generate a library of peptide sequences of specific length and specific offset, to cover the entire native protein sequence NTD IN 1–50 (peptides **1–15**, Figure 1). The solution structure of NTD has been solved by 3D and 2D NMR spectroscopy, classifying NTD as an “all alpha helix” structure. In fact, it consists of three α helices, which range from residues 6–15, 19–25, and 31–39, stabilized by a Zn^2+^ ion coordinated with the HHCC motif (evidenced in Figure 1). Ile^5^, Ala^8^, Ile^28^, Ala^33^, and Ile^36^ form part of the hydrophobic core, which explains their importance for structural integrity (Eijkelenboom et al., 1997). As showed in Figure 1, peptide **2** and peptides **6** and **7** include the 1rd α-helix and 2rd α-helix respectively, while peptide **11** forms the 3rd α-helix of the NTD, and incorporates the Cys^40^ of the HHCC motif.

The relevance of both a α-helix structure and a Zn^2+^ chelating group on the inhibitory activity was assessed by the synthesis of three peptides derived from peptide **11**, in which the Cys residue was deleted (peptide **16**) or substituted by His amino acid a residue also involved in the Zn^2+^ coordination (peptide **17**) (Wolfe et al., 2001; Liao et al., 2013). The same His was also used to replace the C-terminal Ser residue of peptide **16** leading to peptide **18**.

### Biological studies: HIV-1 IN catalytic activity inhibition

The synthetized peptides **1**–**18** were tested *in vitro* for their ability to inhibit the HIV-1 IN. Results showed that peptide **18** was the most potent of the series and inhibit the HIV-1 IN activity with an IC_50_ values of 4.5 μM (Table 1). Peptides **9** and **12** containing the VVAK and VA fragments of peptide **18**, respectively, and peptide **2**, which includes the 6–13 fragment of the first α-helix, maintained a certain inhibitory activity. In this assay, we use Raltegravir as a controls.

**Table 1:** **Sequence, analytical data and effect of peptides 1–18 on HIV-1 IN activities**.

**Peptide**	**Sequence**	**HPLC**	**ESI MS**	**IN inhibition**
		**k'**	**Found**	**IC_50_(μM) ±SD**
**1**	^1^FLDGIDKAQD	4.70	1163.1	NA
**2**	^4^GIDKAQDEHE	5.03	1182.5	162±9
**3**	^7^KAQDEHEKYH	4.63	1326.7	NE
**4**	^10^DEHEKYHSNW	4.75	1385.5	NA
**5**	^13^EKYHSNWRA	5.02	1363.7	NE
**6**	^16^HSNWRAMASD	2.94	1215.5	NE
**7**	^19^WRAMASDFNL	2.30	1251.7	NE
**8**	^22^MASDFNLPPV	1.30	1131.6	NA
**9**	^25^DFNLPPVVAK	3.70	1140.6	213 ± 12
**10**	^28^LPPVVAKEIV	4.40	1105.8	NA
**11**	^31^VVAKEIVASC	2.12	1059.7	NA
**12**	^34^KEIVASCDKC	3.06	1136.5	477 ± 23
**13**	^37^VASCDKCQLK	2.80	1135.5	NA
**14**	^40^CDKCQLKGEA	2.60	1135.4	NA
**15**	^43^CQLKGEAM	3.70	920.3	NA
**16**	^31^VVAKEIVAS	2.50	956.5	NA
**17**	VVAKEIVASH VVAKEIVAH	5.22	1094.0	NA
**18**	VVAKEIVAH	3.50	1007.0	4.5 ± 0.9
**Raltegravir**				0.18 ± 0.01

All peptide are acetylated and amidate at N terminal and C terminal respectively.

k' [(peptide retention time – solvent retention time)/solvent retention time]. Peptide concentration μM.

NE, No effect. The peptide demonstrated no significant effect in the IN assay, where the percent activity for the compounds was found to be 100 ± 7% (As example see the dose-response curve of peptide 5 in Supplementary Material).

NA, Not Applicable, 50% inhibition by the compound was not achieved (As example see the dose-response curve of peptide 8 in Supplementary Material).

Base on the ability of inhibiting HIV-1 IN showed by peptide **18**, we synthesized three peptides, in which Val at position 1, 2, and 7 was substituted with Phe (peptides **19**, **20**, and **21**, respectively). In fact, Phe and Val differ in participating helical assembly of the peptide molecules, and have different hydrophobicity degree (Javadpour and Barkley, 1997; Azmi et al., 2013). In addition, Phe could be involved in additional stacking interactions with the viral target.

Subsequently, we established the contribution of the various amino acid residues to the antiviral activity of peptide **18** (peptides **22–28**) through an *L*-Ala scanning analysis. The results of the biological evaluation of these peptides are summarized in Table 2.

**Table 2:** **Sequence, analytical data, and effect of peptides 19–28 on HIV-1 IN activities**.

**Peptide**	**Sequence**	**HPLC**	**ESI-MS**	**IN inhibition**
		**k'**	**Found**	**IC_50_(μM)**
**18**	VVAKEIVAH	3.50	1007	4.5 ± 0.9
**19**	FVAKEIVAH	4.40	1054.4	NA
**20**	VFAKEIVAH	4.50	1054.5	375 ± 19
**21**	VVAKEIFAH	4.20	1054.2	504 ± 22
**22**	AVAKEIVAH	3.90	978.6	NA
**23**	VAAKEIVAH	4.20	978.4	NA
**24**	VVAAEIVAH	4.84	949.5	20.8 ± 1.3
**25**	VVAKAIVAH	4.05	948.6	13.0 ± 1.0
**26**	VVAKEAVAH	1.39	964.5	NA
**27**	VVAKEIAAH	3.90	978.5	NA
**28**	VVAKEIVAA	4.89	939.5	NA
**Raltegravir**				0.24 ± 0.02

All peptide are acetylated and amidate at C terminal and N terminal respectively.

k' [(peptide retention time – solvent retention time)/solvent retention time]. Peptide concentration μM.

NA = Not Applicable, 50% inhibition by the compound was not achieved (as example see the dose-response curve of peptide 19 in Supplementary Material).

Replacement of the Val residue for the bulky-aromatic Phe led to a strong reduction of the activity of the corresponding peptides **19–21** compared with **18**, indicating the importance of this ramified amino acid on the HIV-1 IN inhibition capability. This result was also confirmed with the data from the Alanine scanning study. In fact, the substitution of Val^1^ or Val^2^ or Val^7^ residues by Ala produced a dramatic loss of the HIV-1 IN inhibitory activity of the corresponding analogs (**22**, **23**, and **27**). The most interesting result was obtained with peptides **24** and **25**. In these cases, the substitution by Ala of Lys^4^ or Glu^5^, respectively, produced only a weak decrease of HIV-1 IN inhibitor ability if compared to the hit peptide, suggesting that a lack of polar side chains in these positions or the introduction of low hindrance, lipophilic features is well accepted.

### Conformational studies

**Figure 2:**
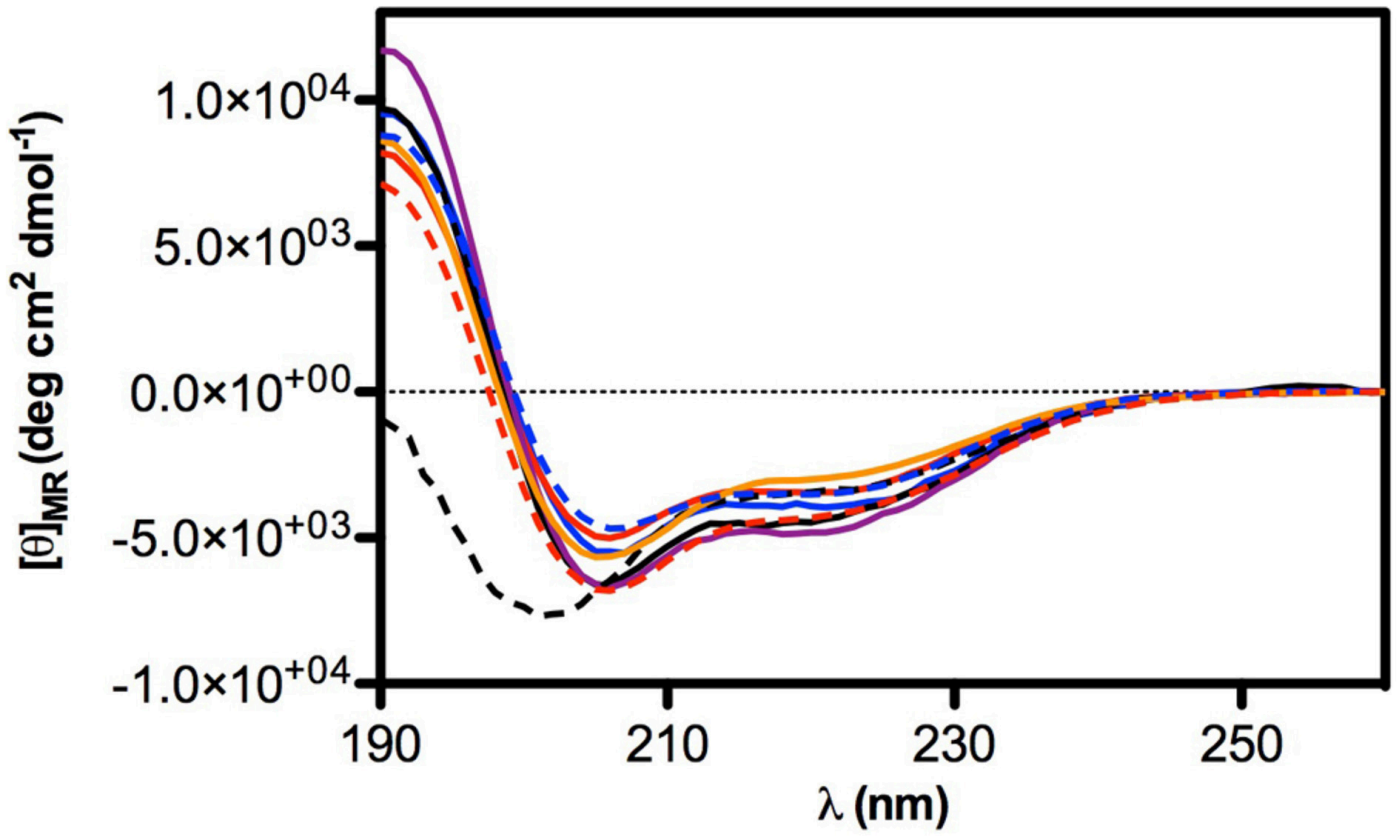
**CD Spectra of selected peptides in TFE/water 50% solution**. Peptide are represented in: **18** (solid blue line), **22** (solid red line), **23** (solid black line), **24** (solid purple line), **25** (dashed black line), **26** (solid orange line), **27** (dashed blue line), **28** (dashed red line).

Circular Dichroism was used to determine the conformational-activity relationships of peptide **18** and its analogs (**22**–**28**). Analysis of spectra acquired in water solution indicated that all peptides are random coil in water (data not shown). In contrast, spectra acquired in 50% TFE/water solution (TFE, trifluoroethanol) indicate that peptides have high tendency to fold as α helix in fluoroalcohol solution (Figure 2). In particular, all peptides have similar high helical content (about 40%) except peptide **25** whose helical content is lower (about 10%).

### IN-IN dimerization and IN multimerization studies

Taking in consideration that these peptides represent fragments of NTD domain, which is involved in different and essential process of the IN activity (Engelman and Craigie, 1992; Cai et al., 1997), we wondered whether they were able to inhibit the IN-IN dimerization or stabilizing the IN in the multimeric form. In fact, it has been reported that allosteric compounds inhibit IN activity binding a site that is different from CCD binding site and working as disruptors of the IN dimer formation (Christ and Debyser, 2013). To verify whether active peptides **18**, **24**, and **25** can modulate the dynamic interplay between IN subunits, we tested them using an HTRF-based IN subunit exchange assay. In this assay two IN preparation are used: a first His-tagged IN preparation, and a second Flag-tagged IN preparation. In both preparations the purified IN is at equilibrium as a dimer; such dimers go through monomers exchange. Hence, in the assay the monomer exchange of the two different preparations leads to the formation of a dimer with one His-tagged subunit and one Flag-tagged subunit. The formation of this “hybrid” dimer can be monitored in the HTRF-based assay as described (Esposito et al., 2015; Tintori et al., 2015). When the assay is performed in the presence of a compound inhibiting dimerization, the HTRF signal decreases as the concentration of compound increases, whereas in the presence of a compound promoting IN multimerization (association of dimers) the HTRF signal increases as the concentration of the compound increases. We found that peptides **24** and **25** modulate the interactions between the IN subunits in different manners using the allosteric inhibitor LEDGIN-6 as a control (Table 3). In fact, peptide **24** was able to inhibit the IN-IN dimer formation with an IC_50_ value of 32 μM, but did not affect IN multimerization, while peptide **25** promoted IN multimerization, with a concentration of compound stimulating the Multimerization Increase by 50% (MI50 value) of 4.8 μM, but did not inhibited IN multimerization. While the reason for this different behavior was not clear, it demonstrates that IN multimerization can be differently modulated by interfering with the NTD sequence. Differently, peptide **18** was not able to modulate the dimer/multimer formation. Hence, the three peptides showed three different modes of IN inhibition in biochemical assays.

**Table 3:** **Effects of peptides 18, 24, and 25 on the HIV-1 IN dimerization and multimerization process**.

**Compounds**	**Sequence**	**IN-IN subunit exchange**	**IN-multim**
		**^a^IC_50_(μM)**	**^c^MI_50_(μM)**
**18**	^31^VVAKEIVAH	>100 (100%)^b^	>100 (100%)
**24**	VVAAEIVAH	32 ± 3	>100 (100%)
**25**	VVAKAIVAH	>100 (100%)	4.8 ± 0.4
**LEDGIN-6**	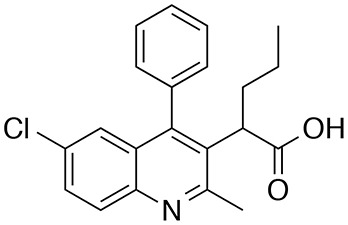	>100 (100%)	10 ± 1

a Compound concentration required to inhibit the HIV-1 IN-IN subunit exchange by 50%.

b Percentage of control measured in the presence of 100 μM concentration.

c Compound concentration required to inhibit the multimerization increase by 50%.

### Cell-based HIV replication assay

The most potent peptides **18**, **24**, and **25**, conveniently conjugated with the cell-penetrating fragment Tat, were evaluated for their capability to inhibit the HIV-1 replication in CD4^+^ MT-4 cells (Table 4). The conjugated peptides **18** and **24** were devoid of antiviral activity when evaluated against HIV replication. In contrast, **25** showed marked inhibitory potency with good selectivity against HIV-1 (IC_50_ value in the low micromolar range, accordingly with the results on IN catalysis and dimerization, and selective index of 5.4), but not against HIV-2.

**Table 4:** **Evaluation of the synthesized peptides against HIV-1 and HIV-2 replication in MT-4 cell cultures**.

**Peptides**	**EC^a^_50_ (μM)**		**CC^b^_50_ (μM)**
	**HIV-1 (NL4.3)**	**HIV-2 (ROD)**	**MT-4**
**Tat-18**	>105	>105	105
**Tat-24**	>50	>50	>50
**Tat-25**	4.0 ± 0.6	>21.4	21.4 ± 2.0

a 50% Effective concentration, or compound concentration required to inhibit HIV-induced cytopathogenic effect in MT-4 cell culture.

b Compound concentration required to reduce by 50% MT-4 cell viability.

## Discussion

A library of peptides was developed from the NTD sequence (1–50) by using a mimotopic strategy. Preliminary results of the *in vitro* integrase assay indicated that the fragment VVAKEIVAH (peptide **18**), which includes a His residue instead of the natural Ser at position 39, reduced significantly the HIV-1 IN activity showing an IC_50_ value of 4.5 μM. Peptide **18** is included into the sequence of the 3rd α-helix of the NTD, which is located on the dimerization interface within the context of the crystal structure of this domain (Cai et al., 1997). Accordingly, we observed significant changes in potency of this peptide when lipophilic and ramified residues involved in the interaction between subunits, such as Val^1^, Val^2^, Ile^6^, and Val^7^ are substituted. Ala scanning showed also that loss of a lysine positively charge or a glutamate negatively charge does not modify substantially the inhibitory activity of the resulting peptides (**24** and **25**, respectively). According to the circular dichroism results, the inhibitory activity of these compounds does not seem to be related to their ability to form helical structures. In fact, inactive peptides **22**, **23**, **26**, **27**, and **28** all show similar helical content as **18** and **24**. On the other hand, compound **25** shows a significant reduction of helical tendency but is approximately as active as **18**. Recently, an approach to develop new HIV-1 IN inhibitors that bind in a pocket different from the active binding site, lead to the discovery of new drugs that are able to interfere with the IN dynamic interplay, in two possible ways (Kessl et al., 2012). In the first one, the molecules interfere with the IN dimer-dimer formation, while the second, the molecules promote the IN multimerization. In this case, IN is trapped in a multimeric form in which the movements of the individual subunits are restricted, and, as a consequence, also the catalytic process. We tested peptides **18**, **24**, and **25** on HTRF assays to determine their potential mechanism of actions and to verify whether they were capable to inhibit the HIV-1 IN. Peptide **24** was able to inhibit the IN-IN dimer formation, while peptide **25** stabilizes the multimeric form of IN. Differently, peptide **18** was not able to modulate the dimer/multimer formation. These data suggest that the modulation of IN dimerization and/or multimerization process could be determined by the net charge of the synthetized peptides, or more specifically by their ability to form intermolecular salt bridges between Glu^5^ (peptide **24**) or Lys^4^ (peptide **25**) residues and Lys or Asp/Glu residues of different subunits, respectively (Michel et al., 2009; Lia et al., 2011). This hypothesis, however, is less probable for peptide **18**, in which the formation of an intramolecular salt bridge between adjacent residues Lys^4^ and Glu^5^ prevents their interactions with acid and basic residues of other subunits. In this case, the *in vitro* inhibitory activity of peptide **18** could be related to its ability of inhibiting IN-DNA interactions.

Interestingly, peptide **25**, conjugated with the cell-penetrating Tat fragment, is the only compound into this series able to inhibit the HIV-1-induced cytopathogenic effect in MT-4 cell. Our results indicate that the presence of a negative charge (Glu^5^ side chain) is detrimental for the inhibition of HIV replication in target CD4 T cells (analogs **18** and **24**), while the positively charged Lys^4^ residue (peptide **25**) plays an important role in the potential antiviral activity. In addition, this peptide has shown to have potent and selective antiviral activity for HIV-1. Although the antiviral activity is about five-fold lower than the concentration of its cellular toxicity, peptide **25** represents a suitable lead compound for the development of novel derivatives with improved toxicity profile.

Overall, our data confirm the hypothesis that peptides derived from the protein of interest may be used to identify domains which are important for catalytic activity, protein−protein interactions, and/or multimerization process (Li et al., 2006; Lia et al., 2011). Data in Tables 1–3 reflect also the difficulties in identifying inhibitor compounds whose biological target is the viral integration process (Liao et al., 2010). In fact, the modification or replacement of a single amino acid in the sequence of the most potent compound in this series (peptide **25)**, determines the diminution or loss of the target activity. The identification of the amino acid residues implicated in the inhibition of IN activity highlights the importance of studies that identify such residues and investigates possible roles for them (Li et al., 2006).

In conclusion, this study shows that the modified 31–39 sequence of the N-terminal domain of HIV integrase (VVAKAIVAH, peptide **25**) is able to interfere with functional integration of the HIV-1 enzyme. This peptide conveniently conjugated (peptide **tat-25**) inhibits the HIV activity in cell culture probably due to its ability to stabilize the IN multimeric form. Our peptide, one of the few in cell active peptides reported to date, could be considered as a suitable hit compound in the development of novel anti-HIV1 peptidomimetic agents with a mechanism of action different to that the approved anti-IN drugs.

## Author contributions

IGM, and PC were responsible for the idea and design of peptides; IGM, PC, MS, ET, and DS designed research; MS, AS, FE, MCS, EV, MC performed research; IGM, PC, ET, DS, MS, AS, FE, MCS, EV, AB, and MM supervised experiments and analyzed data; IGM, MS, AS, MCS, ET, and DS wrote the paper; IGM, PC, PG, AC, EN, ET, FE corrected the manuscript.

## Funding

This work was supported by grants from the Italian Ministry of Education (MIUR) (PRIN n°2010-11E61J12000210001), the FWO (G-485-08 and G.0528.12 N), the KU Leuven (PF/10/018 and GOA/10/014) and the EU FP7 project CHAARM (no. 242135).

### Conflict of interest statement

The authors declare that the research was conducted in the absence of any commercial or financial relationships that could be construed as a potential conflict of interest.

## Abbreviations

IN: integrase
HIV-1: human immunodeficiency virus type 1
PIC: preintegration complex
NTD: N-terminal domain
STIN: strand transfer integrase reaction
CCD: catalytic core domain
CTD: C-terminal domain
INSTIs: integrase strand transfer inhibitors (INSTIs)
HOAt: 1-Hydroxy-7-azabenzotriazole
HOBt: hydroxybenzotriazole
HBTU, N, N, N^*'*^: N^*'*^-tetramethyl-O-(1H-benzotriazol-1-yl)uraniumhexafluorophosphate
DIEA, N: N-diisopropylethylamine
DMF: dimethylformamide.
