# Supplementary material for: Development and Identification of a Novel Anti-HIV-1 Peptide Derived by Modification of the N-Terminal Domain of HIV-1 Integrase
Source: Front Microbiol. 2016 Jun 10;7:845. doi: 10.3389/fmicb.2016.00845 (PMC4901077; doi:10.3389/fmicb.2016.00845)
Supplement: Supplementary file 1 [file DataSheet1.DOC]

**Supporting Information**

**Development and identification of a novel anti-HIV-1 peptide derived by modification of the N-terminal domain of HIV integrase**

Marina Sala^1§^, Antonia Spensiero^1§^, Francesca Esposito^2^, Maria Carmina Scala^1^, Ermelinda Vernieri^1^, Alessia Bertamino^1^, Michele Manfra^3^, Alfonso Carotenuto^4^, Paolo Grieco^4^, Ettore Novellino^4^, Enzo Tramontano^2,5^, Dominique Schols^6^, Pietro Campiglia^1*^, and Isabel M. Gomez-Monterrey^4*^

**Contents**

Figure S1: Dose-response curve of peptide 5.

Figure S2: Dose-response curve of peptide 8.

Figure S3**:** Dose-response curve of peptide 19.

Figure S4: Dose-response curve of peptide 18.

Figure S5: Dose-response curve of peptide 24.

Figure S6: Dose-response curve of peptide 25.

**Figure S1**. Dose-response curve of peptide 5.


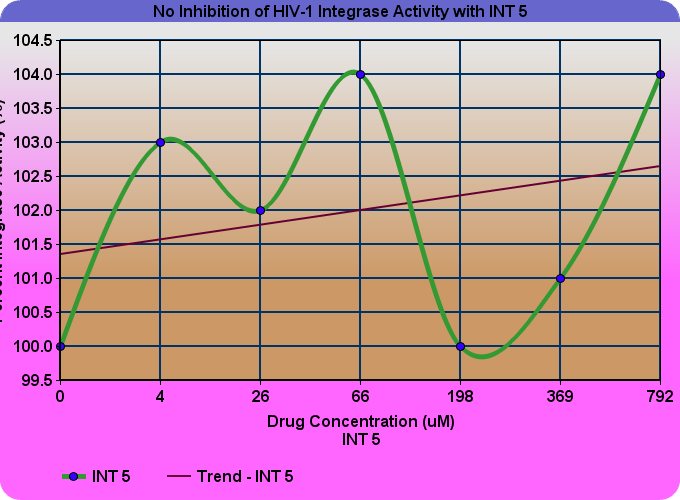


**Figure S2.** Dose-response curve of peptide 8.


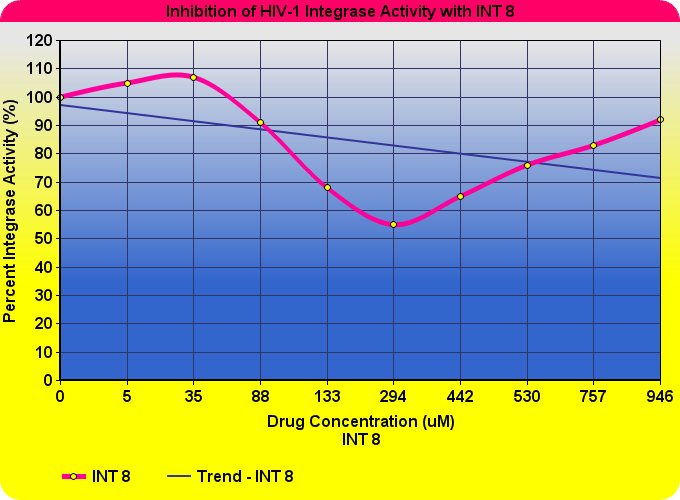


**Figure S3.** Dose-response curve of peptide 19.

**Figure S4.** Dose-response curve of peptide 18.

**Figure S5.** Dose-response curve of peptide 24.

**Figure S6.** Dose-response curve of peptide 26.
